# Dicarbonyl-Dependent Modification of LDL as a Key Factor of Endothelial Dysfunction and Atherosclerotic Vascular Wall Damage

**DOI:** 10.3390/antiox11081565

**Published:** 2022-08-12

**Authors:** Vadim Z. Lankin, Alla K. Tikhaze, Arthur M. Melkumyants

**Affiliations:** Department for Free Radical Research, National Medical Research Center of Cardiology Russian Ministry of Health, 121552 Moscow, Russia

**Keywords:** free radical oxidation, antioxidants, low-density lipoproteins (LDL), oxidatively modified LDL, antioxidant enzymes, LOX-1, glycocalyx, endothelial dysfunction, vascular wall damage, atherosclerosis, diabetes

## Abstract

The review presents evidence that the main damage to the vascular wall occurs not from the action of “oxidized” LDL, which contain hydroperoxy acyls in the phospholipids located in their outer layer, but from the action of LDL particles whose apoprotein B-100 is chemically modified with low molecular weight dicarbonyls, such as malondialdehyde, glyoxal, and methylglyoxal. It has been argued that dicarbonyl-modified LDL, which have the highest cholesterol content, are particularly “atherogenic”. High levels of dicarbonyl-modified LDL have been found to be characteristic of some mutations of apoprotein B-100. Based on the reviewed data, we hypothesized a common molecular mechanism underlying vascular wall damage in atherosclerosis and diabetes mellitus. The important role of oxidatively modified LDL in endothelial dysfunction is discussed in detail. In particular, the role of the interaction of the endothelial receptor LOX-1 with oxidatively modified LDL, which leads to the expression of NADPH oxidase, which in turn generates superoxide anion radical, is discussed. Such hyperproduction of ROS can cause destruction of the glycocalyx, a protective layer of endotheliocytes, and stimulation of apoptosis in these cells. On the whole, the accumulated evidence suggests that carbonyl modification of apoprotein B-100 of LDL is a key factor responsible for vascular wall damage leading to atherogenesis and endothelial dysfunction. Possible ways of pharmacological correction of free radical processes in atherogenesis and diabetogenesis are also discussed.

## 1. Introduction: Free-Radical Oxidation in Atherosclerosis—Background

In the middle of the last century, Denhem Harman proposed the hypothesis that the process of aging is associated with the accumulation of damage at the cellular level, caused by the products of spontaneous free radical oxidation [1,2]. Since such pathologies as atherosclerosis and diabetes mellitus can be attributed to the diseases of old age, D. Harman suggested that the occurrence and development of these pathological conditions (called by him “free radical diseases”) is associated with the damaging effects of free radicals [3]. The first data that free radical peroxidation of lipids may be one of the trigger factors of vascular wall damage in atherosclerosis were published by J. Glavind et al. in 1952 (cited from F.R. Woodford et al. [4]). Based on their results, these authors concluded that the level of lipohydroperoxides (LOOH) in the human aorta with atherosclerotic lesions is higher than in the unaffected vessel wall. In this work, a relatively small number of samples were examined, and analysis based on the dichlorophenol-indophenol method, which was found [4] to be non-specific, was used to estimate the LOOH content. Nevertheless, during the next decade, the conclusions of the work of J. Glavind et al. were not doubted. In as late as 1965, F.R. Woodford et al. [4], using for the analysis of lipoperoxides developed earlier [5] highly specific method of iodometric titration with amperometric registration of the equivalence point, made an attempt to experimentally verify these results. The results obtained by F.R. Woodford et al. practically disproved the conclusion of J. Glavind et al., because these authors failed to identify reliable differences between the content of lipoperoxides in the areas of atherosclerotic lesions and intact aortic areas. The pessimistic conclusions of the publication by F.R. Woodford et al. cooled the interest in the free radical theory of atherogenesis for a long time, despite its theoretical substantiation, given in the articles of D. Harman [1,2,3].

Nevertheless, an increase in free radical peroxidation products was detected in the aorta of animals with experimental atherosclerosis [6]. Two decades later, our group, using an adequate HPLC method, was able to detect a significant increase in the content of primary free radical peroxidation products—LOOH [7] in atherosclerotically damaged aorta, which increased with progression of atherosclerotic lesion [7]. It should be noted that these studies were performed using autopsy material not later than 3 h of those who died in car accidents [7]. It was proved by HPLC on a chiral phase column (by S/R sterioisomer ratio) that LOOH in the atherosclerotically damaged aorta is formed as a result of spontaneous (non-enzymatic) free radical lipoperoxidation [7]. The main class of lipids that accumulate in areas of atherosclerotic lesions of the vascular wall are cholesterol esters [8,9], and not only fatty acid residues are subject to oxidation [6,7], but also the sterol part of their molecule [6,8].

A decrease in the activity of key antioxidant enzymes, Se-containing glutathione peroxidase (GSH-Px) and Cu,Zn-superoxide dismutase (Cu,Zn-SOD), was also observed in the areas of atherosclerotic lesions of human aorta, which progresses with an increasing degree of damage [6,10]. Based on the results obtained, the hypothesis of an imbalance in the action of the systems for the formation and utilization of free radical peroxidation products in tissues in atherosclerosis has been suggested (see reviews [6,11]). The same data provided additional grounds for classifying atherosclerosis as a group of “free radical pathologies”, i.e., diseases in the pathogenesis of which free radical oxidation processes play an important role [6,11]. The main aim of this article is to substantiate the role of dicarbonyl-modified LDL as a key factor of endothelial dysfunction and vascular wall damage in atherosclerosis. 

## 2. Oxidative Stress in Atherosclerosis and Atherogenic Modification of LDL

It should be noted that a significant increase in the levels of primary and secondary products of free radical oxidation of lipids was found in a representative epidemiological study in the blood plasma of probands with diagnosed atherosclerosis [6,11]. In the same study, a decrease in the activity of the LOOH utilizing enzyme, erythrocyte Se-containing glutathione peroxidase (GSH-Px), was found in patients with atherosclerosis [6,11]. These data gave reason to believe that lipid transporting system—blood plasma lipoproteins are subject to oxidation during atherogenesis [6,11]. Indeed, it has been shown that “atherogenic” low-density lipoproteins (LDL) readily undergo oxidation both when incubated in the presence of vascular endotheliocytes and in the presence of free radical oxidation initiators [12,13]. It was found that chemical modification of blood plasma LDL particles by acetaldehyde makes LDL more “atherogenic” [14], i.e., capable of scavenger receptor binding and accumulation in vascular wall macrophages [14]. Later, numerous studies found that LDL particles subjected to free radical peroxidation in vivo or in vitro also become “atherogenic” [15,16,17,18,19,20,21,22].

It is known that free radical peroxidation of lipids proceeds in two stages: first, primary oxidation products are formed (unstable LOOH), which undergo further oxidative degradation with the formation of secondary products—low molecular weight dicarbonyls [23]. Thus, oxidative stress in atherogenesis, characterized by a sharp increase of LOOH in tissues, must inevitably transform into carbonyl stress, accompanied by the accumulation of reactive carbonyl species (RCS) such as 4-hydroxynonenals, malondialdehyde (MDA), etc. [11,23]. Aldehyde groups of dicarbonyls can easily react with the amino end groups of proteins via the Maillard reaction, forming intra- and intermolecular cross-links in their molecules [23]. The possibility of MDA involvement in the modification of apoprotein B-100 LDL has been established [24]; nevertheless, the question about the mechanism of oxidative modification of LDL, due to which LDL particles acquire the property of “atherogenicity”, has not been solved in the literature so far [11]. 

If we stick to the strict terminology, we should call LDL containing hydroperoxy acyls in phospholipids of the outer layer of particles “oxidized”. Basically, the accumulation of hydroperoxy acyls in the phospholipid monolayer of LDL can lead to changes in the conformation of apoprotein B-100. Thus, free radical peroxidation of biomembrane phospholipids increases their microviscosity [6], which increases due to the “pushing out” of more polar hydroperoxy acyls into the aqueous phase [6]. It is very likely that with a significant change in such a fundamental property of bimembranes as microviscosity, the conformation of proteins embedded in the phospholipid bilayer may change. In particular, a multidirectional change in the activity of membrane-bound enzymes was found during free radical peroxidation of liver biomembranes [25], which can be explained by a change in the conformation of these protein molecules with an increase in lipid polarity. Based on these results, one could assume that peroxidation of phospholipids of LDL particles would lead to changes in the conformation of apoprotein B-100, resulting in changes in the binding efficiency of such “oxidized” LDL to the scavenger receptor of macrophages.

When free radical peroxidation of LDL is induced in vitro using a variety of initiators (such as azo-initiators, hydrogen peroxide, superoxide anion radicals, variable-valence metal ions, etc.) almost at the same time both primary (LOOH) and secondary products (MDA and other dicarbonyls) increase [26,27]. On this basis, it is obvious that using standard approaches, it is impossible to establish which lipoperoxidation products cause “atherogenic” modification of LDL particles. Using as a tool C-15 reticulocyte lipoxygenase capable of oxidizing polyene acyls of phospholipids [28], we were able to obtain truly oxidized LDL without an admixture of dicarbonyl-modified LDL [27]. At the same time, during incubation of LDL with MDA, MDA-modified LDL was obtained without an admixture of oxidized (LOOH-containing) LDL [27]. When studying the atherogenicity (the efficiency of the capture of LDL particles by cultured human macrophages) of these two LDL modifications, we experimentally proved that not the “oxidized” (LOOH-containing LDL), but exclusively MDA-modified LDL bind to scavenger receptors of macrophages [27]. Thus, it can be assumed that it is the dicarbonyl-modified LDL particles, but not the truly oxidized (LOOH-containing) LDL, that are efficiently captured by vascular wall cells and accumulate in their lipid vacuoles [27]. As a consequence, macrophages and pericyte-like cells turn into the so-called ‘foam cells’ that form zones of lipoidosis, the primary preatherosclerotic lesions of the vascular wall [6,11,29]. Obviously, the results obtained do not simply clarify the existing terminology, but are of a principled character, since they substantiate the existence of a quite certain molecular mechanism of “atherogenic” modification of LDL particles. Representative epidemiological studies have revealed that the most cholesterol-rich LDL particles are also MDA-modified significantly more often [30]. It follows that dicarbonyl modification of LDL particles contributes to the effective entry of cholesterol into the vascular wall [30]. In addition, there is evidence that increased accumulation of MDA-modified LDL is characteristic of patients with certain mutations of apoB-100, i.e., dicarbonyl modification of LDL can be genetically determined [31].

Cu, Zn-SOD and GSH-Px molecules, similarly to apoprotein B-100 LDL, undergo modification during MDA accumulation during atherogenesis [32,33], which is accompanied by suppression of their activity due to conformational changes in the active center structure [32,33]. It can be assumed that carbonyl-dependent inhibition of the activity of antioxidant enzymes during atherogenesis contributes to the stimulation of oxidative stress. Thus, the development of oxidative and subsequent carbonyl stress (MDA and other RCS accumulation) during atherogenesis leads to the formation of dicarbonyl-modified LDL, which are the key factors causing primary damage to the vascular wall and the subsequent formation of atherosclerotic plaques [11].

## 3. Carbonyl Stress in Diabetes and the Role of Modified LDL in Vascular Wall Damage

Diabetes mellitus is known to be a risk factor for atherosclerosis, and the presence of diabetes contributes to atherogenesis, whereby a large proportion of diabetic patients dying from vascular incidents [34,35,36]. Unfortunately, the available literature does not provide convincing explanations for the cause of these phenomena. Nevertheless, the hypothesis of an important role of free radical peroxidation in the pathogenesis of diabetes mellitus has long been suggested [37]. Diabetes mellitus is characterized by the development of carbonyl stress rather than oxidative stress [38], which accumulates not MDA, but active dicarbonyls formed during oxidative transformations of glucose, such as glyoxal and methylglyoxal [38,39,40]. Glyoxal is formed at glyoxylation during autooxidation of glucose and other six-atom carbohydrates, whereas methylglyoxal is synthesized during glycolysis during enzymatic oxidation of glucose from triosophosphates [38,41,42]. However, as shown in our studies, methylglyoxal can also be formed nonenzymatically when glucose derivatives are attacked by peroxy free radicals [43]. 

When LDL cooxidation in the presence of glucose in concentrations characteristic for blood levels of patients with type 2 diabetes, there is a sharp increase in the rate of LDL lipid peroxidation, which is accompanied by the formation of superoxide anion radical [44]. Superoxide radical can also be generated during the Maillard reaction at the interaction of methylglyoxal with the end amino groups of LDL apoprotein B-100 [43]. Thus, in diabetogenesis, in contrast to atherogenesis, carbonyl stress (accumulation of RCS) develops initially, and oxidative stress has a secondary origin and is induced at later stages by reactive oxygen species (ROS) generated due to the reactions described above. On this basis, during diabetogenesis a distinction must be made between stages of carbonyl stress and subsequent oxidative stress, which are characterized by the accumulation of various oxidation products. The accumulation of glyoxal and methylglyoxal in the plasma of diabetic patients has been repeatedly confirmed [38,39,40]. At the same time, the presence of oxidative stress in diabetes is evidenced by a decrease in telomere length of nuclear blood cells [45], as well as an increase in the level of 8-hydroxy-2-deoxyguanosine, the end product of oxidative DNA destruction, in the blood and urine of type 2 diabetic patients [45]. It should be noted that 8-hydroxy-2-deoxyguanosine is a recognized biomarker of oxidative stress [46] and its accumulation is not associated with the development of carbonyl stress. The presence of elevated levels of LOOH-containing LDL [38] in the blood of type 2 diabetic patients also indicates that secondary induction of oxidative stress may indeed occur during atherogenesis.

Just as in atherosclerosis, in patients with type 2 diabetes there is an increase in the carbonyl modification of LDL [45] and a sharp decrease in the activity of erythrocyte Cu,Zn-SOD and GSH-Px [45,47], which is a reflection of carbonyl stress. A significant increase in glyoxal and methylglyoxal levels in the blood of type 2 diabetic patients [38,39,40] can cause modification of LDL, which is detected by scavenger receptor macrophages and, thus, can induce the accumulation of LDL in the vascular wall with the subsequent development of lipoid lesions [38]. It was shown that LDL modification by methylglyoxal significantly increases LDL “atherogenicity” (increases their receptor uptake by macrophages) [38,48]. Based on the above data, we hypothesized a common molecular mechanism of vascular wall damage in atherosclerosis and diabetes mellitus, which includes an increase in chemical modification of apoB-100 LDL by dicarbonyls accumulated during free radical lipid peroxidation in atherosclerosis or autooxidation of glucose molecules in diabetes mellitus [44]. This hypothesis satisfactorily explains the reasons for the stimulation of atherogenesis in diabetes, as well as the fact that the presence of diabetes may increase the risk of atherosclerosis [44]. 

## 4. Oxidatively Modified LDL, LOX-1 and Endothelial Dysfunction

In recent years, it has been found that oxidized LDL can play an important role in the occurrence of endothelial dysfunction [49,50,51,52]. It is assumed that the endotheliocyte scavenger receptor LOX-1 binds to oxidized LDL, causing the expression of NADPH oxidase, which generates superoxide anion radicals and other ROS, causing endothelial cell damages [53]. We received preliminary data showing that powerful expression of LOX-1 and NADPH oxidase in human endotheliocytes is caused by cell culturing in the presence of different dicarbonyl-modified (MDA-, glyoxal- and methylglyoxal-modified) LDLs [54]. Consequently, the initial stages of vascular endothelial dysfunction, a process that plays a leading role in atherogenesis and diabetogenesis, are likely to be directly dependent on the formation of dicarbonyl-modified LDL rather than “oxidized” LDL. Ultimately, superoxide-dependent endotheliocyte damage provokes the stimulation of apoptosis and endothelial cell death [49,52,53], which, in turn, obviously facilitates the penetration of modified LDL into the vascular wall.

We found that the enzyme antioxidant system of endotheliocytes is represented mainly by a special class of enzymes, peroxiredoxins [55], which, in accordance with our data, similar to Cu,Zn-SOD and GSH-Px [32,47], are very sensitive to the inhibitory action of low molecular weight dicarbonyls that accumulate under oxidative and carbonyl stress [56]. Obviously, the suppression of peroxiredoxin activity weakens the antiradical protection of endothelial cells, contributing to endothelial damage, leading to its dysfunction. Thus, the data obtained suggest that the formation of dicarbonyl-modified LDL is a key factor in atherosclerosis and endothelial dysfunction development, processes that play a leading role in atherogenesis and diabetogenesis.

## 5. Active Oxygen Species and Glycocalyx Degradation

It is clear that endothelial dysfunction must be preceded by endothelial glycocalyx damage. Glycocalyx is a protective layer of macromolecules (such as proteoglycans and glycoproteins) covering the luminal surface of endotheliocytes [57,58]. Its damage is considered as the earliest stage of vascular wall injury in various pathologies [59,60,61,62]. Glycocalyx controls the permeability of the vascular wall [63] and adhesion of blood forming elements to endotheliocytes [64,65]. Besides, glycocalyx protects endothelium from such damaging factors as viruses, proinflammatory cytokines and ROS [66,67]. It is very important that glycocalyx macromolecules are mechanoreceptors which perceive the force of viscous friction (shear stress) acting on the vascular wall from the flowing blood, which is the first stage in regulation of vascular hydraulic resistance according to blood flow. This regulation ensures normal blood supply of the organs, counteraction of constriction and acute phase of collateral blood supply development in case of occlusion of the main arterial trunks [68]. The regulation of vascular tone according to shear stress is more expressed the thicker the glycocalyx layer is. At the same time, the thickness of glycocalyx depends on the shear stress value: the higher it is, the thicker the glycocalyx layer is. Therefore, the areas of vessels characterized by low shear stress and, consequently, a thin glycocalyx layer occurs in the areas where the first signs of atherosclerotic lesions appear and further atheromatous plaques are formed [69].

This can be explained by the fact that the glycocalyx layer is the main barrier preventing the penetration of atherogenic LDL (obviously, oxidatively modified LDL) into the subendothelial space of the vessel wall [70]. Glycocalyx thinning occurs due to a decrease in the content of its main components—hyaluronan and heparan sulfate [71], whose biosynthesis rate by endotheliocytes decreases at low shear stress [69]. A decrease in glycocalyx thickness due to its fragmentation has been noted under ROS hyperproduction during oxidative stress in the process of ischemia and ischemia/reperfusion [72,73,74] and a degradation of glycosaminoglycans by ROS derived from stimulated polymorphonuclear leukocytes has been detected [75]. Glycocalyx destruction was also observed when the level of oxidized LDL in plasma increased [76,77]. We found that endothelium-dependent regulation of arterial lumen is suppressed by an increase of MDA and/or methylglyoxal content in blood [78]. These facts indicate that oxidatively modified LDL (as follows from the above most probably dicarbonyl-modified LDL) formed under oxidative stress and/or carbonyl stress are the most important factors of atherogenesis. Consequently, glycocalyx preservation should prevent atherogenesis and diabetogenesis, and glycocalyx damage can be regarded as the first stage of atherosclerotic vascular injury. It may be assumed that intensification of free radical processes during atherogenesis and diabetogenesis, provoking oxidative modification of LDL, in addition may trigger other molecular mechanisms of atherosclerotic damage of the vascular wall, such as glycocalyx degradation by superoxide anion radicals and other ROS.

**Figure 1:**
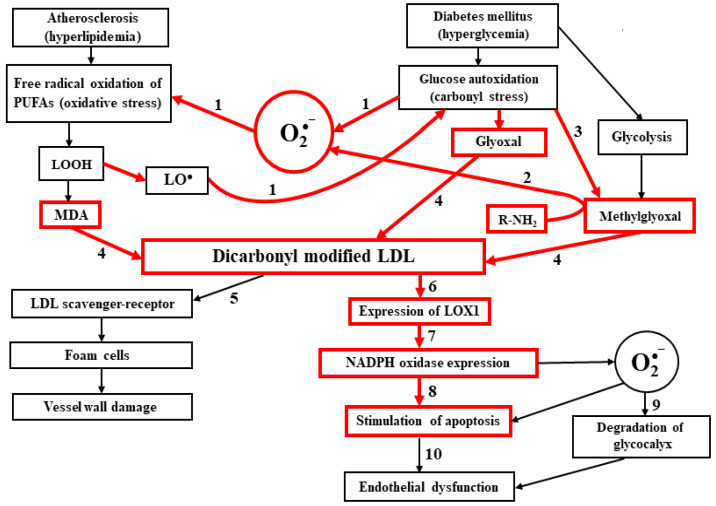
The main stages of atherogenic damage of the vascular wall during oxidative and carbonyl stress in atherogenesis and diabetogenesis and the development of endothelial dysfunction under the action of dicarbonyl-modified LDL (based on literature and own data). Red lines and rectangles highlight the metabolic reactions and processes whose existence was experimentally confirmed by the authors of this review. Numbers denote: 1. Pathways of secondary development of oxidative stress in diabetes associated with increased free-radical mechanism of endotheliocyte membrane damage and development of endothelial dysfunction lipoperoxidation in atherogenesis [42,44]; 2. An alternative pathway of oxidative stress development in diabetes associated with the formation of superoxide anion radical and other ROS during the reaction of amino-containing compounds with methylglyoxal (Maillard reaction) [43]; 3. Non-enzymatic formation of methylglyoxal when glucose derivatives are attacked by alkoxyl lipid radicals [43]; 4. Chemical modification of LDL, involving various natural dicarbonyls: MDA, glyoxal, and methylglyoxal [11,24,27,38]; 5. Foam cell formation and preaterosclerotic (lipoidosis) damage of the vascular wall under the action of dicarbonyl-modified LDL [11]; 6. Expression of LOX-1 on endotheliocyte membranes under the action of dicarbonyl-modified LDL [54]; 7. Expression of NADPH-oxidase in endotheliocytes during the accumulation of dicarbonyl-modified LDL and under generation of superoxide anion radical by this enzyme system [54]; 8. Stimulation of endotheliocyte apoptosis by superoxide anion radical and other ROS generated by NADPH-oxidase [50,51,52,53]; 9. Glycocalyx degradation under the action of superoxide anion radical and other ROS [71,72,73,74,75]; 10. Free-radical mechanism of endotheliocyte membrane damage and development of endothelial dysfunction [49,50,51,52,53].

Based on a review of the cited literature and our own data, we can propose the following scheme, which summarizes current views on the molecular mechanisms of atherogenic damage of the vascular wall and endothelial dysfunction (Figure 1).

## 6. Antioxidants in Cardiology: Pro Et Contra

Based on the above considerations, it is logical to use antioxidants to inhibit LDL oxidation, and a number of clinical studies have attempted to use safe natural antioxidants, such as vitamin E (α-tocopherol, α-TOH), for this purpose. Trail data on the intervention of antioxidants (mainly α-TOH, in some cases combined with ascorbate and/or β-carotene) in cardiovascular diseases, in contrast to the very encouraging positive results obtained from animal studies with experimental atherosclerosis, are not so unequivocal [79,80,81,82,83]. A number of randomized, double blind, placebo-controlled studies have found that the use of antioxidant vitamins significantly reduces cardiovascular risk and cardiac mortality [84,85,86], and one of the few studies in which angiography was used as a control [87] documented suppression of coronary stenosis in patients treated with antioxidants [87]. The works executed on large contingents of men [88] and women [89] have demonstrated that regular consumption of α-TOH for several years promotes the reliable reduction of risk of occurrence of CHD [88,89]. In the Cambridge Heart Antioxidant Study (CHAOS), more than 2000 patients with angiographically confirmed atherosclerosis received high (400–800 IU/day) doses of α-TOH for a year, and there was a significant reduction in the risk of myocardial infarction [90]. In the SPACE study, in which patients with CHD underwent hemodialysis and therapy including 800 IU/day of α-TOH for almost 1.5 years, there was a significant reduction in the incidence of myocardial infarction [91]. However, a number of other clinical trials did not show a significant reduction in cardiovascular complications or cardiac mortality when antioxidants were administered [92,93,94,95]. For example, in a study including a large number of male smokers who took α-TOH and/or β-carotene for 5–8 years, there was no significant increase in cardiovascular mortality [92]. In the GISSI-Prevenzione trial, administration of 450 IU/day of α-TOH to patients who had no more than 3 months after myocardial infarction resulted in neither a reduction in mortality, nor a decrease in the incidence of new infarcts or strokes within 3.5 years [93]. In the Heart Outcomes Prevention Evaluation Study (HOPE), over 1500 patients at high risk of cardiovascular disease who received 400 IU/day of α-TOH for 4.5 years showed no significant reduction in cardiovascular mortality [94]. In the MRC/BHF Heart Protection Study, over 20,000 patients with CHD who received an antioxidant vitamin complex that included 900 IU/day of α-TOH for 5 years showed no increase in mortality from heart attacks and strokes [95].

The results of studies using antioxidants (which included extremely high doses of α-TOH), in which, contrary to expectations, no clear positive results were obtained, have led some authors to argue that the antioxidants used had a negative effect [79,80]. Obviously, it is not correct to interpret the lack of effect as a negative effect. Of course, the ambiguity of the results on the use of antioxidants in clinical trials raises the question of the need for a critical analysis of the reasons for this. At the same time, it should be noted that none of the studies cited above reliably established a “negative” effect of antioxidants (e.g., increased mortality and/or cardiac complications), as they revealed only the absence of the expected positive effect. Based on the design of the studies, the principles of selection of the used antioxidants and their doses, the criteria for assessing biochemical and clinical changes, it seems obvious that the results of such very expensive works a priori cannot give an unambiguous answer to the questions posed in them. We can agree with the statements of opponents of further research on the use of antioxidants in cardiology about the futility of continuing such research [79,80] without new evidence-based approaches to planning and conducting the work.

It should be noted that the choice of α-TOH (vitamin E) as an antioxidant used in most of the above studies cannot be considered sufficiently successful and justified. α-TOH, like other fat-soluble vitamins, is transported in the body as part of the hydrophobic lipid core of LDL particles [96]. However, it is not α-TOH that protects circulating LDL particles from free radical peroxidation in the bloodstream, but the reduced (phenolic) form of coenzyme Q [6,97,98,99,100,101]. Based on the fact that in one particle of LDL, there are no more than 1–2 coenzyme Q molecules per 800 molecules of free radical peroxidation substrate, unsaturated phospholipids [102,103] effective inhibition of free radical reactions in LDL by this antioxidant is impossible without its bioregeneration, possibly by mechanism with the participation of α-TOH and ascorbate [103,104,105,106,107]. At the same time, it has been shown that administration of α-TOH at high doses has no effect on LDL oxidizability in CHD patients in vivo [101]. Thus, it should be recognized that the use of α-TOH to inhibit LDL oxidizability in clinical trials is not justified and the use of coenzyme Q [6,100] and other phenolic antioxidants, in particular probucol [6,101,108,109,110], whose effectiveness in inhibiting LDL oxidation is convincingly confirmed, is more correctly for LDL protection against oxidation [6,101]. It is also clear that it is unacceptable to use data about the “negative” results obtained with individual antioxidants, such as α-TOH and β-carotene [92,93,94], for the entire group of antioxidants [80], which includes a large number of substances with very different structures and mechanisms of action. Although α-TOH is safe in doses tens of times greater than its daily requirement [111], the use of extremely high doses of natural antioxidants can be dangerous because of the possibility of converting the antioxidant action into a pro-oxidant action, as we showed in an in vivo study [112].

Moreover, the data presented in this review indicate that in order to suppress atherogenesis and endothelial dysfunction it is necessary to inhibit not only (and perhaps not so much) the accumulation of primary products (LOOH) in LDL, but also the accumulation of secondary products of free radical oxidation—low molecular weight dicarbonyls. Positive examples of influencing the intensity of free radical oxidation using biguanides—dicarbonyl scavengers [101,113,114,115] and aldehyde-binding imidazole-containing peptides [116,117] already exist. In particular, the use of biguanides significantly suppressed the manifestation of oxidative and carbonyl stress in diabetic patients without the administration of any antioxidants (the so-called “quasi-antioxidant effect”) [101]. Obviously, preventive cardiology should be aimed at preventing the negative effects of oxidative modification of LDL, since modified LDL play a key role in the molecular mechanisms of atherogenesis and diabetogenesis. Currently, the urgent task of pharmacotherapy is to develop effective approaches to drug suppression of the accumulation of primary and secondary products of free radical oxidation (as well as ROS and RCS) in order to control the level of potentially dangerous oxidized and modified LDL.

## 7. Conclusions: Dicarbonyl-Modification LDL Is a Key Factor of Endothelial Dysfunction and Atherogenesis

Experimental evidence is reviewed that not “oxidized” LDL (LDL containing LOOH acyls in phospholipids), but LDL particles whose apoprotein B-100 is chemically modified with natural dicarbonyls (such as malonic dialdehyde, glyoxal and methylglyoxal) may be responsible for vascular wall damage. Based on the literature data and our own results, we hypothesized a common molecular mechanism of vascular wall lesions in atherosclerosis and diabetes mellitus. Oxidatively modified LDL also plays an important role in endothelial dysfunction: the LOX-1 receptor of these cells binds to oxidatively modified LDL, causing expression of NADPH oxidase and generation of superoxide anion radicals, which causes endothelial cell damage and apoptosis. Consequently, carbonyl modification of LDL may be a key factor in vascular wall injury in atherogenesis and endothelial dysfunction.

## References

[1] Harman D. (1956). Aging: A Theory Based on Free Radical and Radiation Chemistry. J. Gerontol..

[2] Harman D. (1984). Free radical theory of aging: The “free radical” diseases. Age.

[3] Harman D. (2003). The Free Radical Theory of Aging. Antioxid. Redox Signal..

[4] Woodford F., Böttcher C., Oette K., Ahrens E. (1965). The artifactual nature of lipid peroxides detected in extracts of human aorta. J. Atheroscler. Res..

[5] Oette K., Peterson M.L., McAuley R.L. (1963). A highly sensitive method for measurement of lipid hydroperoxides by iodometry and amperometric endpoint. J. Lipid Res..

[6] Lankin V.Z., Tikhaze A.K. (2003). Atherosclerosis as a free radical pathology and antioxidative therapy of this disease. Free Radicals, NO and Inflammation.

[7] Kühn H., Belkner J., Wiesner R., Schewe T., Lankin V.Z., Tikhaze A.K. (1992). Structure elucidation of oxygenated lipids in human atherosclerotic lesions. Eicosanoids.

[8] Harland W., Gilbert J.D., Brooks C.J. (1973). Lipids of human atheroma: VIII. Oxidised derivatives of cholesteryl linoleate. Biochim. Biophys. Acta.

[9] Carpenter K.L., Taylor S.E., Ballantine J.A., Fussell B., Halliwell B., Mitchinson M.J. (1993). Lipids and oxidised lipids in human atheroma and normal aorta. Biochim. Biophys. Acta.

[10] Lankin V.Z., Vikhert A.M., Kosykh V.A., Tikhaze A.K., Galakhov I.E., Orekhov A.N., Repin V.N. (1984). Enzymatic detoxication of superoxide anion-radical and lipoperoxides in intima and media of atherosclerotic aorta. Biomed. Biochim. Acta.

[11] Lankin V., Tikhaze A. (2017). Role of Oxidative Stress in the Genesis of Atherosclerosis and Diabetes Mellitus: A Personal Look Back on 50 Years of Research. Curr. Aging Sci..

[12] Esterbauer H., Gebicki J., Puhl H., Jürgens G. (1992). The role of lipid peroxidation and antioxidants in oxidative modification of LDL. Free Radic. Biol. Med..

[13] Steinbrecher U.P., Parthasarathy S., Leake D.S., Witztum J.L., Steinberg D. (1984). Modification of low density lipoprotein by endothelial cells involves lipid peroxidation and degradation of low density lipoprotein phospholipids. Proc. Natl. Acad. Sci. USA.

[14] Goldstein J.L., Ho Y.K., Basu S.K., Brown M.S. (1979). Binding site on macrophages that mediates uptake and degradation of acetylated low density lipoprotein, producing massive cholesterol deposition. Proc. Natl. Acad. Sci. USA.

[15] Epstein F.H., Steinberg D., Parthasarathy S., Carew T.E., Khoo J.C., Witztum J.L. (1989). Beyond Cholesterol. Modification of low density lipoprotein that increase its atherogenicity. N. Engl. J. Med..

[16] Steibrecher U.P., Lougheed M., Kwan W.-C., Dirks M. (1989). Recognition of oxidized low density lipoprotein by the scavenger receptor of macrophages results from derivatization of apoprotein B by products fatty acid peroxidation. J. Biol. Chem..

[17] Kita T., Ishii K., Yokode M., Kume N., Nagano Y., Arai H., Kawai C. (1990). The role of oxidized low density lipoprotein in the pathogenesis of atherosclerosis. Eur. Heart J..

[18] Witztum J.L., Steinberg D. (1991). Role of oxidized low density lipoprotein in atherogenesis. J. Clin. Investig..

[19] Witztum J. (1994). The oxidation hypothesis of atherosclerosis. Lancet.

[20] Ylä-Herttuala S. (1991). Macrophages and Oxidized Low Density Lipoproteins in the Pathogenesis of Atherosclerosis. Ann. Med..

[21] Yla-Herttuala S. (1994). Role of lipid and lipoprotein oxidation in the pathogenesis of atherosclerosis. Drugs Today.

[22] Steinberg D. (1995). Role of Oxidized LDL and Antioxidants in Atherosclerosis. Adv. Exp. Med. Biol..

[23] Estévez M., Padilla P., Carvalho L., Martín L., Carrapiso A., Delgado J. (2019). Malondialdehyde interferes with the formation and detection of primary carbonyls in oxidized proteins. Redox. Biol..

[24] Fogelman A.M., Shechter I., Seager J., Hokom M., Child J.S., Edwards P.A. (1980). Malondialdehyde alteration of low density lipoproteins leads to the cholesteryl ester accumulation in human monocyte-macrophages. Proc. Natl. Acad. Sci. USA.

[25] Lankin V.Z. (1980). Lipid peroxides and atherosclerosis. Hypothesis: The role of cholesterol and free-radical lipid peroxidation in altering cell membrane properties in hypercholesterolemia and atherosclerosis. Kardiologiia.

[26] Halliwell B. (2006). Reactive Species and Antioxidants. Redox Biology Is a Fundamental Theme of Aerobic Life. Plant Physiol..

[27] Lankin V.Z., Tikhaze A.K., Kumskova E.M. (2012). Macrophages actively accumulate malonyldialdehyde-modified but not enzymatically oxidized low density lipoprotein. Mol. Cell. Biochem..

[28] Schewe T., Rapoport S.M., Kühn H. (1986). Enzymology and Physiology of Reticulocyte Lipoxygenase: Comparison with Other Lipoxygenases. Adv. Enzymol..

[29] Summerhill V., Orekhov A. (2019). Pericytes in Atherosclerosis. Adv. Exp. Med. Biol..

[30] Lankin V., Viigimaa M., Tikhaze A., Kumskova E., Konovalova G., Abina J., Zemtsovskaya G., Kotkina T., Yanushevskaya E., Vlasik T. (2011). Cholesterol-rich low density lipoproteins are also more oxidized. Mol. Cell. Biochem..

[31] Khlebus E., Kutsenko V., Meshkov A., Ershova A., Kiseleva A., Shcherbakova N., Zharikova A., Drapkina O., Shevtsov A., Yarovaya E. (2019). Multiple rare and common variants in APOB gene locus associated with oxidatively modified low-density lipoprotein levels. PLoS ONE.

[32] Tikhaze A.K., Kosach V.Y., Lankin V.Z., Panferova A.A., Smirnova M.D. (2020). Indicator Characterizing Carbonyl-Dependent Modification of Erythrocytic Superoxydismutase as a Biochemical Marker of Oxidative Stress in Coronary Heart Disease. Kardiologiia.

[33] Lankin V.Z., Shumaev K.B., Tikhaze A.K., Kurganov B.I. (2017). Influence of dicarbonyls on kinetic characteristics of glutathione peroxidase. Dokl. Biochem. Biophys..

[34] Nishizawa T., Bornfeldt K.E. (2012). Diabetic vascular disease and the potential role of macrophage glucose metabolism. Ann. Med..

[35] Bornfeldt K.E. (2016). Does Elevated Glucose Promote Atherosclerosis? Pros and Cons. Circ. Res..

[36] Poznyak A., Grechko A.V., Poggio P., Myasoedova V.A., Alfieri V., Orekhov A.N. (2020). The Diabetes Mellitus–Atherosclerosis Connection: The Role of Lipid and Glucose Metabolism and Chronic Inflammation. Int. J. Mol. Sci..

[37] Oberley L.W. (1988). Free radicals and diabetes. Free Radic. Biol. Med..

[38] Lankin V.Z., Tikhaze A.K., Kapel’Ko V.I., Shepel’Kova G.S., Shumaev K.B., Panasenko O.M., Konovalova G.G., Belenkov Y.N. (2007). Mechanisms of oxidative modification of low density lipoproteins under conditions of oxidative and carbonyl stress. Biochemistry.

[39] Thornalley P.J., Langborg A., Minhas H.S. (1999). Formation of glyoxal, methylglyoxal and 3-deoxyglucosone in the glycation of proteins by glucose. Biochem. J..

[40] Wang X.-J., Ma S.-B., Liu Z.-F., Li H., Gao W.-Y. (2019). Elevated levels of α-dicarbonyl compounds in the plasma of type II diabetics and their relevance with diabetic nephropathy. J. Chromatogr. B Analyt. Technol. Biomed. Life. Sci..

[41] Spiteller G. (2005). The relation of lipid peroxidation processes with atherogenesis: A new theory on atherogenesis. Mol. Nutr. Food Res..

[42] Spiteller G. (2008). Peroxyl Radicals Are Essential Reagents in the Oxidation Steps of the Maillard Reaction Leading to Generation of Advanced Glycation End Products. Ann. N. Y. Acad. Sci..

[43] Lankin V., Shadyro O., Shumaev K., Tikhaze A., Sladkova A. (2019). Non-Enzymatic Methylglyoxal Formation From glucose Metabolites and Generation of Superoxide Anion Radical during Methylglyoxal-Dependent Cross-Links Reaction. J. Antioxid. Act..

[44] Lankin V., Konovalova G., Tikhaze A., Shumaev K., Kumskova E., Viigimaa M. (2014). The initiation of free radical peroxidation of low-density lipoproteins by glucose and its metabolite methylglyoxal: A common molecular mechanism of vascular wall injure in atherosclerosis and diabetes. Mol. Cell. Biochem..

[45] Lankin V.Z., Tikhaze A.K., Konovalova G.G., Odinokova A.O., Doroshchuk A.N., Chazova E.I. (2018). Oxidative and carbonyl stress as factors of protein modification and DNA destruction in diabetes mellitus. Ther. Arch..

[46] Graille M., Wild P., Sauvain J.-J., Hemmendinger M., Canu I.G., Hopf N.B. (2020). Urinary 8-OHdG as a Biomarker for Oxidative Stress: A Systematic Literature Review and Meta-Analysis. Int. J. Mol. Sci..

[47] Lankin V.Z., Konovalova G.G., Tikhaze A.K., Shumaev K.B., Kumskova E.M.B., Grechnikova M.A., Viigimaa M. (2016). Aldehyde inhibition of antioxidant enzymes in the blood of diabetic patients. J. Diabetes.

[48] Knott H.M., Brown B.E., Davies M.J., Dean R.T. (2003). Glycation and glycoxidation of low-density lipoproteins by glucose and low-molecular mass aldehydes. Formation of modified and oxidized particles. Eur. J. Biol. Chem..

[49] Pirillo A., Norata G.D., Catapano A.L. (2013). LOX-1, OxLDL, and Atherosclerosis. Mediat. Inflamm..

[50] Lubrano V., Balzan S. (2014). LOX-1 and ROS, inseparable factors in the process of endothelial damage. Free Radic. Res..

[51] Chistiakov D.A., Orekhov A.N., Bobryshev Y.V. (2016). LOX-1-Mediated Effects on Vascular Cells in Atherosclerosis. Cell. Physiol. Biochem..

[52] Kattoor A.J., Kanuri S.H., Mehta J.L. (2019). Role of Ox-LDL and LOX-1 in Atherogenesis. Curr. Med. Chem..

[53] Galle J., Schneider R., Heinloth A., Wanner C., Galle P.R., Conzelmann E., Dimmeler S., Heermeier K. (1999). Lp(a) and LDL induce apoptosis in human endothelial cells and in rabbit aorta: Role of oxidative stress. Kidney Int..

[54] Lankin V.Z., Sharapov M.G., Goncharov R.G., Antonova O.A., Tikhaze A.K., Konovalova G.G. (2022). Expression of LOX-1 and NADPH Oxidase in Endotheliocytes by Dicarbonyl-Modified LDL.

[55] Sharapov M.G., Goncharov R.G., Gordeeva A.E., Novoselov V.I., Antonova O.A., Tikhaze A.K., Lankin V.Z. (2016). Enzymatic antioxidant system of endotheliocytes. Dokl. Biochem. Biophys..

[56] Lankin V.Z., Sharapov M.G., Goncharov R.G., Tikhaze A.K., Novoselov V.I. (2019). Natural Dicarbonyls Inhibit Peroxidase Activity of Peroxiredoxins. Dokl. Biochem. Biophys..

[57] Weinbaum S., Tarbell J.M., Damiano E.R. (2007). The Structure and Function of the Endothelial Glycocalyx Layer. Annu. Rev. Biomed. Eng..

[58] Reitsma S., Slaaf D.W., Vink H., van Zandvoort M.A.M.J., Oude Egbrink M.G. (2007). The endothelial glycocalyx: Composition, functions, and visualization. Pflug. Arch..

[59] Noble M., Drake-Holland A., Vink H. (2008). Hypothesis: Arterial glycocalyx dysfunction is the first step in the atherothrombotic process. QJM.

[60] Becker B.F., Jacob M., Leipert S., Salmon A.H.J., Chappell D. (2015). Degradation of the endothelial glycocalyx in clinical settings: Searching for the sheddases. Br. J. Clin. Pharmacol..

[61] Pillinger N.L., Kam P.C.A. (2017). Endothelial Glycocalyx: Basic Science and Clinical Implications. Anaesth. Intensive Care.

[62] Nieuwdorp M., van Haeften T.W., Gouverneur M.C., Mooij H.L., van Lieshout M.H., Levi M., Meijers J.C., Holleman F., Hoekstra J.B., Vink H. (2006). Loss of Endothelial Glycocalyx during Acute Hyperglycemia Coincides with Endothelial Dysfunction and Coagulation Activation In Vivo. Diabetes.

[63] Curry F.E., Adamson R.H. (2012). Endothelial Glycocalyx: Permeability Barrier and Mechanosensor. Ann. Biomed. Eng..

[64] Mulivor A.W., Lipowsky H.H. (2002). Role of glycocalyx in leukocyte-endothelial cell adhesion. Am. J. Physiol.-Heart Circ. Physiol..

[65] Egbrink M.G.A.O., Heijnen V.V.T., Megens R.T.A., Engels W., Vink H., Slaaf D.W., van Zandvoort M.A.M.J., Reitsma S. (2011). Endothelial glycocalyx thickness and platelet-vessel wall interactions during atherogenesis. Thromb. Haemost..

[66] Alphonsus C.S., Rodseth R. (2014). The endothelial glycocalyx: A review of the vascular barrier. Anaesthesia.

[67] Henrich M., Gruss M., Weigand M.A. (2010). Sepsis-Induced Degradation of Endothelial Glycocalix. Sci. World J..

[68] Melkumyants A.M., Balashov S.A., Khayutin V.M. (1995). Control of arterial lumen by shear stress on endothelium. NIPS.

[69] Gouverneur M., Berg B., Nieuwdorp M., Stroes E., Vink H. (2006). Vasculoprotective properties of the endothelial glycocalyx: Effects of fluid shear stress. J. Intern. Med..

[70] Berg B.M.V.D., Spaan J.A.E., Vink H. (2009). Impaired glycocalyx barrier properties contribute to enhanced intimal low-density lipoprotein accumulation at the carotid artery bifurcation in mice. Pflüg. Arch.-Eur. J. Physiol..

[71] Dogné S., Flamion B. (2020). Endothelial Glycocalyx Impairment in Disease: Focus on hyaluronan shedding. Am. J. Pathol..

[72] Rehm M., Bruegger D., Christ F., Conzen P., Thiel M., Jacob M., Chappell D., Stoeckelhuber M., Welsch U., Reichart B. (2007). Shedding of the Endothelial Glycocalyx in Patients Undergoing Major Vascular Surgery with Global and Regional Ischemia. Circulation.

[73] Chappell D., Jacob M., Hofmann-Kiefer K., Rehm M., Welsch U., Conzen P., Becker B.F. (2009). Antithrombin reduces shedding of the endothelial glycocalyx following ischaemia/reperfusion. Cardiovasc. Res..

[74] Rubio-Gayosso I., Platts S.H., Duling B.R. (2006). Reactive oxygen species mediate modification of glycocalyx during ischemia-reperfusion injury. Am. J. Physiol.-Heart Circ. Physiol..

[75] Moseley R., Waddington R., Embery G. (1997). Degradation of glycosaminoglycans by reactive oxygen species derived from stimulated polymorphonuclear leukocytes. Biochim. Biophys. Acta.

[76] Vink H., Constantinescu A.A., Spaan J.A.E. (2000). Oxidized Lipoproteins Degrade the Endothelial Surface Layer: Implications for platelet-endothelial cell adhesion. Circulation.

[77] Constantinescu A.A., Vink H., Spaan J.A.E. (2001). Elevated capillary tube hematocrit reflects degradation of endothelial cell glycocalyx by oxidized LDL. Am. J. Physiol.-Heart Circ. Physiol..

[78] Ermishkin V.V., Lukoshkova E.V., Melkumyants A.M. (2021). Malonyldialdehyde- and Methylglyoxal-Induced Suppression of Endothelium-Mediated Dilation of Rat Iliac Artery in Response to Elevation of Blood Flow. J. Evol. Biochem. Physiol..

[79] Jialal I., Traber M., Devaraj S. (2001). Is there a vitamin E paradox?. Curr. Opin. Lipidol..

[80] Kuller L.H. (2001). A Time to Stop Prescribing Antioxidant Vitamins to Prevent and Treat Heart Disease?. Arter. Thromb. Vasc. Biol..

[81] Steinberg D. (2000). Is there a potential therapeutic role for vitamin E or other antioxidants in atherosclerosis?. Curr. Opin. Lipidol..

[82] Witztum J.L., Steinberg D. (2001). The oxidative modification hypothesis of atherosclerosis: Does it hold for humans?. Trends Cardiovasc. Med..

[83] Steinberg D., Witztum J.L. (2002). Is the Oxidative Modification Hypothesis Relevant to Human Atherosclerosis? Do the antioxidant trials conducted to date refute the hypothesis?. Circulation.

[84] Losonczy K.G., Harris T.B., Havlik R.J. (1996). Vitamin E and vitamin C supplement use and risk of all-cause and coronary heart disease mortality in older persons: The Established Populations for Epidemiologic Studies of the Elderly. Am. J. Clin. Nutr..

[85] Steinberg D. (1993). Antioxidant Vitamins and Coronary Heart Disease. N. Engl. J. Med..

[86] Steinberg D. (1995). Clinical trials of antioxidants in atherosclerosis: Are we doing the right thing?. Lancet.

[87] Hodis H.N., Mack W.J., La Bree L., Cashin-Hemphill L., Sevanian A., Johnson R., Azen S.P. (1995). Serial Coronary Angiographic Evidence That Antioxidant Vitamin Intake Reduces Progression of Coronary Artery Atherosclerosis. JAMA.

[88] Rimm E.B., Stampfer M.J., Ascherio A., Giovannucci E., Colditz G.A., Willett W.C. (1993). Vitamin E Consumption and the Risk of Coronary Heart Disease in Men. N. Engl. J. Med..

[89] Stampfer M.J., Hennekens C.H., Manson J.E., Colditz G.A., Rosner B., Willett W.C. (1993). Vitamin E Consumption and the Risk of Coronary Disease in Women. N. Engl. J. Med..

[90] Stephens N., Parsons A., Brown M., Schofield P., Kelly F., Cheeseman K., Mitchinson M. (1996). Randomised controlled trial of vitamin E in patients with coronary disease: Cambridge Heart Antioxidant Study (CHAOS). Lancet.

[91] Boaz M., Smetana S., Weinstein T., Matas Z., Gafter U., Iaina A., Knecht A., Weissgarten Y., Brunner D., Fainaru M. (2000). Secondary prevention with antioxidants of cardiovascular disease in endstage renal disease (SPACE): Randomised placebo-controlled trial. Lancet.

[92] Alpha-Tocopherol, Beta Carotene Cancer Prevention Study Group (1994). The Effect of Vitamin E and Beta Carotene on the Incidence of Lung Cancer and Other Cancers in Male Smokers. N. Engl. J. Med..

[93] Gruppo Italiano per lo Studio della Sopravvivenza nell’Infarto Miocardico (1999). Dietary supplementation with n-3 polyunsaturated fatty acids and vitamin E after myocardial infarction: Results of the GISSI-Prevenzione trial. Lancet.

[94] Yusuf S., Dagenais G., Pogue J., Bosch J., Sleight P. (2000). Vitamin E Supplementation and Cardiovascular Events in High-Risk Patients. The Heart Outcomes Prevention Evaluation Study Investigators. N. Engl. J. Med..

[95] Heart Protection Study Collaborative Group (2002). MRC/BHF Heart Protection Study of antioxidant vitamin supplementation in 20,536 high-risk individuals: A randomised placebo-controlled trial. Lancet.

[96] Traber M., Burton G., Ingold K., Kayden H. (1990). RRR- and SRR-alpha-tocopherols are secreted without discrimination in human chylomicrons, but RRR-alpha-tocopherol is preferentially secreted in very low density lipoproteins. J. Lipid Res..

[97] Bowry V., Ingold K.U., Stocker R. (1992). Vitamin E in human low-density lipoprotein. When and how this antioxidant becomes a pro-oxidant. Biochem. J..

[98] Stocker R., Bowry V.W., Frei B. (1991). Ubiquinol-10 protects human low density lipoprotein more efficiently against lipid peroxidation than does α-tocopherol. Proc. Natl. Acad. Sci. USA.

[99] Mohr D., Bowry V.W., Stocker R. (1992). Dietary supplementation with coenzyme Q10 results in increased levels of ubiquinol-10 within circulating lipoproteins and increased resistance of human low-density lipoprotein to the initiation of lipid peroxidation. Biochim. Biophys. Acta.

[100] Ahmadvand H., Mabuchi H., Nohara A., Kobayahi J., Kawashiri M.-A. (2013). Effects of coenzyme Q(10) on LDL oxidation in vitro. Acta Med. Iran..

[101] Lankin V.Z., Tikhaze A.K., Kukharchuk V.V., Konovalova G.G., Pisarenko O.I., Kaminnyi A.I., Shumaev K.B., Belenkov Y.N. (2003). Antioxidants decreases the intensification of low density lipoprotein free radical peroxidation during therapy with statins. Mol. Cell. Biochem..

[102] Stocker R. (1993). Natural antioxidants and atherosclerosis. Asia Pac. J. Clin. Nutr..

[103] Frei B., Kim M.C., Ames B.N. (1990). Ubiquinol-10 is an effective lipid-soluble antioxidant at physiological concentrations. Proc. Natl. Acad. Sci. USA.

[104] Beyer R.E. (1994). The role of ascorbate in antioxidant protection of biomembranes: Interaction with vitamin E and coenzyme Q. J. Bioenerg. Biomembr..

[105] Packer J.E., Slater T.F., Willson R.L. (1979). Direct observation of a free radical interaction between vitamin E and vitamin C. Nature.

[106] Niki E., Saito T., Kawakami A., Kamiya Y. (1984). Inhibition of oxidation of methyl linoleate in solution by vitamin E and vitamin C. J. Biol. Chem..

[107] Tikhaze A.K., Konovalova G.G., Lankin V.Z., Kaminnyi A.I., Kaminnaja V.I., Ruuge E.K., Kukharchuk V.V. (2005). Effect of Ubiquinone Q10 and Antioxidant Vitamins on Free Radical Oxidation of Phospholipids in Biological Membranes of Rat Liver. Bull. Exp. Biol. Med..

[108] Kagan V.E., Freisleben H.-J., Tsuchiya M., Forte T., Packer L. (1991). Generation of Probucol Radicals and Their Reduction by Ascorbate and Dihydrolipoic Acid in Human Low Density Lipoproteins. Free Radic. Res. Commun..

[109] Shumaev K.B., Ruuge E.K., Dmitrovsky A.A., Bykhovsky V.Y., Kukharchuk V.V. (1997). Effect of lipid peroxidation products and antioxidants on the formation of probucol radical in low density lipoproteins. Biochemistry.

[110] Tikhaze A.K., Lankin V.Z., Konovalova G.G., Shumaev K.B., Kaminnyi A.I., Kozachenko A.I., Gurevich S.M., Nagler L.G., Zaitseva T.M., Kukharchuk V.V. (1999). Antioxidant probucol as an effective scavenger of lipid radicals in low density lipoproteins in vivo and in vitro. Bull. Exp. Biol. Med..

[111] Simons L.A., Von Konigsmark M., Balasubramaniam S. (1996). What dose of vitamin E is required to reduce susceptibility of LDL to oxidation?. Aust. N. Z. J. Med..

[112] Lankin V.Z., Tikhaze A.K., Konovalova G.G., Kozachenko A.I. (1999). Concentration inversion of the antioxidant and pro-oxidant effects of beta-carotene in tissues in vivo. Bull. Eksp. Biol. Med..

[113] Ruggiero-Lopez D., Lecomte M., Moinet G., Patereau G., Lagarde M., Wiernsperger N. (1999). Reaction of metformin with dicarbonyl compounds. possible implication in the inhibition of advanced glycation end product formation. Biochem. Pharmacol..

[114] Beisswenger P., Ruggiero-Lopez D. (2003). Metformin inhibition of glycation processes. Diabetes Metab..

[115] Wang G., Wang Y., Yang Q., Xu C., Zheng Y., Wang L., Wu J., Zeng M., Luo M. (2022). Metformin prevents methylglyoxal-induced apoptosis by suppressing oxidative stress in vitro and in vivo. Cell Death Dis..

[116] Boldyrev A.A., Aldini G., Derave W. (2013). Physiology and Pathophysiology of Carnosine. Physiol. Rev..

[117] Reddy V.P., Garrett M.R., Perry G., Smith M.A. (2005). Carnosine: A Versatile Antioxidant and Antiglycating Agent. Sci. Aging Knowl. Environ..

